# Efficacy and Safety of Ombitasvir/Paritaprevir/Ritonavir and Dasabuvir With or Without Ribavirin in Patients With Chronic Hepatitis C Virus Genotype 1 Infection Receiving Opioid Substitution Therapy: A Post Hoc Analysis of 12 Clinical Trials

**DOI:** 10.1093/ofid/ofy248

**Published:** 2018-09-27

**Authors:** Jason Grebely, Massimo Puoti, Heiner Wedemeyer, Curtis Cooper, Mark S Sulkowski, Graham R Foster, Thomas Berg, Erica Villa, Federico Rodriguez-Perez, David L Wyles, Gretja Schnell, Negar N Alami, Zhenzhen Zhang, Emily Dumas, Gregory J Dore

**Affiliations:** 1 The Kirby Institute, UNSW Sydney, Sydney, Australia; 2 Azienda Ospedaliera Ospedale Niguarda Ca’ Granda, Milan, Italy; 3 Medizinische Hochschule Hannover, Hannover, Germany; 4 Faculty of Medicine, University of Ottawa, Ottawa, Ontario, Canada; 5 Viral Hepatitis Center, Johns Hopkins University, Baltimore, Maryland; 6 Queen Mary University of London and Barts Health NHS Trust, London, United Kingdom; 7 Universitätsklinikum Leipzig, Leipzig, Germany; 8 University of Modena and Reggio Emilia, Modena, Italy; 9 Gastroenterology Department, VA Caribbean Healthcare System, San Juan, Puerto Rico; 10 Infectious Diseases Division, Department of Medicine at Denver Health, Denver, Colorado; 11 AbbVie Inc., North Chicago, Illinois

**Keywords:** drug use, hepatitis C virus, opioid substitution therapy, PWID

## Abstract

**Background:**

We evaluated the impact of opioid substitution therapy (OST) on the completion, adherence, efficacy, and safety of the 3-direct-acting antiviral regimen of ombitasvir, paritaprevir (identified by AbbVie and Enanta) co-dosed with ritonavir, and dasabuvir ± ribavirin among patients infected with hepatitis C virus (HCV) genotype (GT) 1, with or without compensated cirrhosis.

**Methods:**

Data were pooled from GT1-infected patients enrolled in 12 phase II/III/IIIb clinical trials and categorized by use of OST. Patients with ongoing drug use were excluded. HCV treatment completion, treatment adherence (≥90%), sustained virologic response at post-treatment week 12 (SVR12), and adverse events were assessed.

**Results:**

Of 4747 patients, 3% (n = 149) received OST. Among patients receiving OST vs those not receiving OST, 82% (n = 122) vs 52% (n = 2409) had GT1a infection; 76% (n = 113) vs 61% (n = 2792) were treatment naïve; and 17% (n = 25) vs 18% (n = 830) had cirrhosis, respectively. The proportion of patients completing HCV treatment did not differ between those receiving and not receiving OST (97% [n = 144] vs 98% [n = 4510], respectively), whereas adherence to treatment was reduced in patients receiving vs those not receiving OST (88% [n = 105] vs 97% [n = 4057], respectively). SVR12 was similar between patients receiving and not receiving OST (94% [n = 140] vs 96% [n = 4405], respectively; *P* = .273). Treatment was well tolerated.

**Conclusions:**

Although treatment adherence was lower in patients receiving OST vs those not receiving OST, treatment completion and SVR12 were similar between groups. These data support the use of direct-acting antiviral therapies in patients receiving OST.

In many countries, the majority of new and existing cases of hepatitis C virus (HCV) infection occur among people who inject drugs (PWID), currently or formerly [1]. This includes people receiving opioid substitution therapy (OST) for the management of opioid dependence. The availability of simple and well-tolerated direct-acting antiviral (DAA) therapies with the ability to cure >95% of patients offer an unprecedented opportunity to reverse the rising burden of advanced liver disease among people receiving OST [2]. However, in some settings, people receiving OST are still ineligible [3] or might not be considered suitable by practitioners [4] to receive DAAs. Concerns about treatment adherence and poorer outcomes than in people not receiving OST, and the potential risk of re-infection are often cited as reasons for not treating HCV in people receiving OST [5].

Post hoc analyses of phase II and III trials of DAA therapy have demonstrated that sustained virologic response (SVR) is similar in those receiving and not receiving OST [6–13]. In the first dedicated clinical trial of DAA therapy among people receiving OST, the all-oral 3-DAA regimen of ombitasvir, paritaprevir with the pharmacokinetic enhancer ritonavir, and dasabuvir in combination with ribavirin was evaluated for 12 weeks in 38 participants receiving methadone (n = 19) or buprenorphine (n = 19) OST with HCV genotype (GT) 1 (84% GT1a) without cirrhosis [14]. Participants with a positive urine drug screen for noncannabis drugs at screening were excluded. Overall, 97% (37/38) of participants in the study completed treatment, and 97% (37/38) achieved sustained virologic response at post-treatment week 12 (SVR12), with no participants experiencing virologic failure. However, further data on HCV treatment outcomes in people receiving OST are needed.

Ombitasvir, paritaprevir with the pharmacokinetic enhancer ritonavir, and dasabuvir with or without ribavirin is approved for the treatment of patients with HCV GT1 infection. In clinical trials, ombitasvir/paritaprevir/ritonavir and dasabuvir with or without ribavirin demonstrated an SVR12 of ≥94% across many patient groups, including those receiving OST [12–22].

The aim of this post hoc analysis was to evaluate the impact of OST on treatment completion, adherence, SVR, and safety of ombitasvir/paritaprevir/ritonavir and dasabuvir with or without ribavirin in patients with chronic HCV GT1 infection with or without decompensated cirrhosis.

## METHODS

### Study Participants and Design

From September 10, 2012, to April 28, 2016, participants were enrolled in 12 international phase II, phase III, and phase IIIb studies including SAPPHIRE-I/II (ClinicalTrials.gov identifiers: NCT01716585 and NCT01715415) [12, 13], PEARL-II/III/IV (NCT01674725, NCT01767116, and NCT01833533) [15, 16], TURQUOISE-I/II/III (NCT01939197, NCT01704755, and NCT02219503) [17–19], TOPAZ-VA (NCT02442284) [20], TOPAZ-I/II (NCT02219490 and NCT02167945) [21, 22], and NCT01911845 [14].

Participants with chronic HCV GT1 without cirrhosis were treated with ombitasvir/paritaprevir/ritonavir and dasabuvir with or without ribavirin for 12 weeks, and patients with compensated cirrhosis were treated with ombitasvir/paritaprevir/ritonavir and dasabuvir with ribavirin for 12 or 24 weeks or without ribavirin for 12 weeks [12–22].

Participants receiving OST (eg, methadone or buprenorphine) were eligible for inclusion in 6 of the studies included in the analysis. Patients with ongoing drug use were generally excluded from these trials based on the results of urine drug screens or at the investigator’s discretion [12–22]. Study designs and patient inclusion/exclusion criteria have been described previously [12–22].

### Study End Points

In this analysis, the end points included treatment completion, adherence (≥90% of doses), SVR12, safety (adverse events [AEs], laboratory abnormalities, and serious AEs), and re-infection. The analysis population included all randomized patients who received ≥1 dose of ombitasvir/paritaprevir/ritonavir and dasabuvir with or without ribavirin (intent-to-treat analysis). Treatment completion was defined as ≥77 days of treatment for 12-week regimens and ≥154 days for 24-week regimens. Adherence was calculated by dividing the number of total pills received during therapy (determined by pill counts at study visits in weeks 4, 8, 12, and 24 [where applicable]) by the total expected number of pills. Patients with missing adherence data due to incomplete record of pill counts were excluded from adherence analyses. SVR12 was defined as HCV RNA less than the lower limit of quantification of 25 IU/mL in serum, measured by the COBAS TaqMan HCV Test, v2.0 (Roche Molecular Systems), at post-treatment week 12. Participants were monitored for relapse/re-infection for 24 to 104 weeks following the completion of treatment. Phylogenetic analyses were used to distinguish virologic failure from re-infection.

### Statistical Analysis

Descriptive statistics, including means, frequencies, and percentages (with 95% confidence intervals for SVR12), were used to summarize the data. The proportion of participants with treatment completion, ≥90% adherence, SVR12, and AEs were compared among people receiving and not receiving OST. All HCV GT1 subtypes other than GT1a/1b were categorized and analyzed as GT1a. Comparisons were made using the chi-square test to assess the difference of proportions between 2 groups, except for the comparison between adherence to ribavirin and DAA (ombitasvir/paritaprevir/ritonavir and dasabuvir), for which a logistic regression model with random effects was used for the paired binary data with missing values. All *P* values are 2-sided; a level of .05 was considered statistically significant. Confidence intervals for SVR12 were constructed using the Wilson score method for a binomial proportion. Statistical analysis was performed using SAS 9.2 software (SAS Institute Inc., Cary, NC).

## RESULTS

### Participant Characteristics

Of the 4747 patients enrolled and treated across these 12 clinical trials, 3% (n = 149) were receiving OST at enrollment. The demographic baseline and clinical characteristics of the study participants are shown in Table 1. Compared with those not receiving OST, people receiving OST were younger (median age, 49 years vs 54 years) and more often male (75% vs 57%), infected with HCV GT1a (82% vs 52%), and treatment naïve (76% vs 61%). The proportion with cirrhosis was similar in those receiving and not receiving OST (17% vs 18%, respectively).

**Table 1.** Baseline Demographic and Clinical Characteristics of Patients With Chronic HCV Genotype 1 Receiving Ombitasvir/Paritaprevir/Ritonavir and Dasabuvir With or Without Ribavirin, Stratified by Receipt of Opioid Substitution Therapy

Characteristic	Receiving OST (n = 149)	Not Receiving OST (n = 4598)
Male, No. (%)	112 (75)	2639 (57)
Caucasian, No. (%)	140 (94)	4177 (91)
Age, median (range), y	49 (19–69)	54 (18–83)
BMI, median (range), kg/m^2^	26 (16–45)	26 (16–52)
Geographic region, No. (%)		
North America	92 (62)	1968 (43)
Europe	53 (36)	2453 (53)
Australia/New Zealand	0	44 (1)
Asia	0	51 (1)
Tobacco use (current), No. (%)^a^	108 (72)	1367 (30)
Alcohol use (current), No. (%)^b^	37 (25)	1368 (30)
History of injecting drug use, No. (%)^c^		
Yes	58 (39)	859 (19)
No	10 (7)	1592 (35)
Missing	81 (54)	2142 (47)
HCV GT1 subtype, No. (%)		
1a	122 (82)	2409 (52)
1b	27 (18)	2189 (48)
*IFNL3 (IL28B*) non-CC genotype, No. (%)^d^	98 (66)	3568 (78)
Baseline HCV ≥800 000 log_10_ IU/mL, No. (%)^e^	114 (77)	3526 (77)
Treatment naïve, No. (%)	113 (76)	2792 (61)
HIV infection, No. (%)	18 (12)	271 (6)
Fibrosis stage, No. (%)^f^		
F0–F1	84 (56)	2599 (57)
F2	22 (15)	618 (13)
F3	18 (12)	547 (12)
F4	25 (17)	830 (18)
Therapy		
DAA + RBV	138 (93)	3071 (67)
DAA	11 (7)	1527 (33)

Abbreviations: BMI, body mass index; DAA, direct-acting antiviral; GT, genotype; HCV, hepatitis C virus; *IL28B*, interleukin-28B gene; OST, opioid substitution therapy; RBV, ribavirin.

^a^Tobacco use was unknown for 20 patients (0.4%) who were not receiving OST.

^b^Alcohol use was unknown for 1 (0.7%) and 54 patients (1%) among those receiving OST and those not receiving OST, respectively.

^c^Former injection drug use information was not collected in 2 of the 12 studies included in this analysis.

^d^ *IFNL3* genotype was missing for 1 (0.7%) and 20 patients (0.4%) among those receiving OST and those not receiving OST, respectively.

^e^Baseline HCV RNA was missing for 3 patients (0.07%) who were not receiving OST.

^f^Baseline fibrosis stage was missing for 4 patients (0.09%) who were not receiving OST. Fibrosis stage was determined using the results of a liver biopsy, FibroScan, or FibroTest/aspartate aminotransferase to platelet ratio index.

Among people receiving OST, methadone was prescribed in 50% (75/149), buprenorphine alone or with naloxone in 40% (59/149), and other unspecified OST medications in 10% (15/149). Among people receiving OST, 14% (n = 21) and 68% (n = 101) were infected with HCV GT1a with and without compensated cirrhosis, respectively, and 3% (n = 4) and 15% (n = 23) were infected with HCV GT1b with and without compensated cirrhosis, respectively.

### HCV Treatment Completion

The proportion of patients completing HCV therapy was 97% (144/149) in participants receiving OST compared with 98% (4510/4598) in those not receiving OST (*P* = .211) (Table 2). For the 5 participants receiving OST who discontinued therapy, the reasons for discontinuation included AEs (n = 3) and loss to follow-up (n = 2). Among the 89 participants not receiving OST who discontinued therapy, the reasons for discontinuation included AEs (n = 32), virologic failure (n = 13), noncompliance (n = 5), withdrawn consent (n = 16), loss to follow-up (n = 10), and other (n = 13).

**Table 2.** Treatment Completion, Adherence, and Summary of SVR12 and Reasons for Non-SVR12 Among Patients With Chronic HCV Genotype 1 Receiving Ombitasvir/Paritaprevir/Ritonavir and Dasabuvir With or Without Ribavirin, Stratified by Receipt of Opioid Substitution Therapy

	Receiving OST	Not Receiving OST	*P* Value
Treatment completion, n/N (%)^a^	144/149 (97)	4510/4598 (98)	.211
Treatment adherence, n/N (%)^b^			
Overall treatment^c^	105/120 (88)	4057/4173 (97)	<.001
DAA + RBV			
DAA	114/124 (92)	2855/2893 (99)	<.001
RBV	101/113 (89)	2699/2780 (97)	<.001
DAA only			
DAA	11/11 (100)	1437/1465 (98)	.643
SVR12, n/N (%)	140/149 (94)	4405/4598 (96)	.273
Reasons for non-SVR12, No. (%)			
On-treatment virologic failure	0	25 (0.5)	
Premature study drug discontinuation (adverse events)	2 (1)	17 (0.4)	
Premature study drug discontinuation (lost to follow-up)	2 (1)	8 (0.2)	
Virological relapse	2 (1)	75 (2)	
Lost to follow-up following completion of treatment (SVR data missing)	3 (2)	48 (1)	

Abbreviations: DAA, direct-acting antiviral (ombitasvir/paritaprevir/ritonavir + dasabuvir); HCV, hepatitis C virus; OST, opioid substitution therapy; RBV, ribavirin; SVR12, sustained virologic response at post-treatment week 12.

^a^Treatment completion was defined as ≥77 days of treatment for 12-week regimens and ≥154 days for 24-week regimens.

^b^Adherence was calculated by dividing the number of total pills received during therapy (determined by pill counts at study visits in weeks 4, 8, 12, and 24 [where applicable]) by the total expected number of pills.

^c^Overall treatment adherence included people who had complete adherence data for DAA + RBV therapy (both DAA and RBV) and DAA therapy.

### HCV Treatment Adherence

The proportion of participants with ≥90% adherence to all study treatment was 88% (105/120) in those receiving OST compared with 97% (4057/4173) in those not receiving OST (*P* < .001) (Table 2). For participants receiving OST and ombitasvir/paritaprevir/ritonavir plus dasabuvir with ribavirin, adherence to ribavirin was similar to adherence with ombitasvir/paritaprevir/ritonavir plus dasabuvir (89% vs 92%; *P* = .517). Adherence to ribavirin was lower than adherence to the DAAs in participants not receiving OST (97% vs 99%; *P* < .001). Among participants receiving ribavirin-free therapy, the proportion with ≥90% adherence to therapy was 100% (11/11) for those receiving OST compared with 98% (1437/1465) in those not receiving OST (Table 2).

### HCV Treatment Outcomes

**Figure 1.**
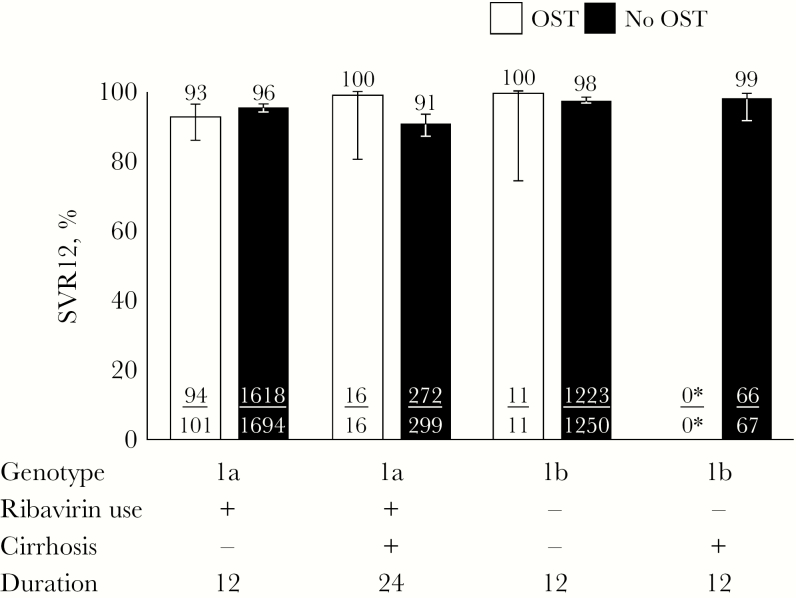
SVR12 in patients receiving and not receiving OST by label-recommended ombitasvir/paritaprevir/ritonavir and dasabuvir with or without ribavirin. Error bars represent 95% confidence intervals. ^*^No genotype 1b-infected patients with cirrhosis receiving OST received the label-recommended therapy. Abbreviations: OST, opioid substitution therapy; SVR12, sustained virologic response at post-treatment week 12.

The proportion of participants with end-of-treatment response among all those receiving ombitasvir/paritaprevir/ritonavir and dasabuvir with or without ribavirin was 97% (145/149) in those receiving OST compared with 98% (4523/4598) in those not receiving OST (*P* = .323). The proportion of participants with SVR12 among all those receiving ombitasvir/paritaprevir/ritonavir and dasabuvir with or without ribavirin was 94% (140/149) in those receiving OST compared with 96% (4405/4598) in those not receiving OST (*P* = .273). The reasons for non-SVR in both those receiving and not receiving OST are shown in Table 2. The SVR12 by label-recommended ombitasvir/paritaprevir/ritonavir and dasabuvir with or without ribavirin is shown in Figure 1.

### Safety

There were no serious DAA-related AEs in patients receiving OST, and the proportions of DAA-related AEs leading to discontinuation of the study drug were low and similar among patients receiving OST and those not receiving OST (Table 3). Frequencies of AEs were generally comparable between patients receiving OST vs those not receiving OST; nausea and anxiety were the only 2 AEs occurring with a frequency ≥5% higher in patients receiving OST vs those not receiving OST (Table 4).

**Table 3.** Safety Summary Among Patients With Chronic HCV Genotype 1 Receiving Ombitasvir/Paritaprevir/Ritonavir and Dasabuvir With or Without Ribavirin, Stratified by Receipt of Opioid Substitution Therapy and Receipt of Ribavirin

No. (%)	Receiving OST			Not Receiving OST		
	All (n = 149)	No RBV (n = 11)	+ RBV (n = 138)	All (n = 4598)	No RBV (n = 1527)	+ RBV (n = 3071)
Any AE	127 (85)	9 (82)	118 (85)	3596 (78)	991 (65)	2605 (85)
DAA-related AE	98 (66)	6 (55)	92 (67)	2613 (57)	666 (44)	1947 (63)
Serious AE	3 (2)	0	3 (2)	132 (3)	27 (2)	105 (3)
DAA-related serious AE	0	0	0	20 (<1)	4 (<1)	16 (1)
AE leading to D/C	3 (2)	0	3 (2)	37 (1)	5 (<1)	32 (1)
DAA-related AE leading to D/C	2 (1)	0	2 (1)	26 (1)	2 (<1)	24 (1)
Death	2 (1)	1 (10)	1 (1)	16 (<1)	4 (<1)	12 (<1)

Abbreviations: AE, adverse event; DAA, direct-acting antiviral (ombitasvir/paritaprevir/ritonavir + dasabuvir); D/C, discontinuation; HCV, hepatitis C virus; OST, opioid substitution therapy; RBV, ribavirin.

**Table 4.** Adverse Events Occurring in ≥10% of Patients With Chronic HCV Genotype 1 Receiving Ombitasvir/Paritaprevir/Ritonavir and Dasabuvir With or Without Ribavirin, Stratified by Receipt of Opioid Substitution Therapy

No. (%)	Receiving OST (n = 149)	Not Receiving OST (n = 4598)
Fatigue	41 (28)	1264 (28)
Headache	29 (20)	1035 (23)
Nausea	39 (26)	690 (15)
Pruritus	17 (11)	560 (12)
Insomnia	21 (14)	547 (12)
Diarrhea	10 (7)	466 (10)
Rash	15 (10)	276 (6)
Anxiety	16 (11)	172 (4)

Abbreviations: HCV, hepatitis C virus; OST, opioid substitution therapy.

### HCV Re-infection

HCV re-infection occurred in 7/4747 (0.2%) participants included in this analysis who received at least 1 dose of ombitasvir/paritaprevir/ritonavir and dasabuvir with or without ribavirin. There was 1 case of HCV re-infection in the period following the end of treatment among 149 participants receiving OST. This patient relapsed 104 weeks after treatment and was determined to be re-infected with an isolate of GT1a that was genetically distinct from the one present at baseline before treatment. Six cases of HCV re-infection were also observed among 4598 participants not receiving OST, of which 5 patients with HCV GT1a infection at baseline were re-infected with a genetically distinct isolate of GT1a during the post-treatment period. One patient infected with GT1b at baseline was re-infected with HCV GT3a at post-treatment week 52. Three cases of re-infection occurred in patients with HIV-1/HCV coinfection, 2 of whom reported high-risk behaviors of unprotected anal intercourse with a sexual partner after completion of study drug treatment [19]. Among the 7 cases of HCV re-infection, all re-infections occurred during the post-treatment period between 8 and 104 weeks after the end of treatment (n = 1 at 8 weeks post-treatment, 2 at 12 weeks post-treatment, 1 at 24 weeks post-treatment, 1 at 52 weeks post-treatment, and 2 at 104 weeks post-treatment).

## DISCUSSION

In this post hoc analysis of data from 12 international clinical trials, there was no difference observed in the proportion of patients who completed HCV treatment or achieved SVR12 among people receiving and not receiving OST treated with ombitasvir/paritaprevir/ritonavir and dasabuvir with or without ribavirin. However, the proportion with ≥90% adherence to therapy was significantly lower in patients receiving OST compared with those not receiving OST. Treatment was also well tolerated. This study provides additional data to support DAA treatment for people receiving OST, consistent with international guidelines [23–26].

Overall, 97% of people receiving OST completed therapy in this study, which was similar to the response in those not receiving OST (98%). These data are also consistent with other post hoc studies demonstrating that 96%–97% of people receiving OST and DAA therapy completed therapy in phase III clinical trials of sofosbuvir/ledipasvir and sofosbuvir/velpatasvir [7, 8]. This is also similar to data from a large phase III study of elbasvir/grazoprevir therapy, in which 99% of patients receiving opioid agonist therapy completed DAA therapy, perhaps further facilitated by the use of a daily adherence reminder [11]. Despite a high proportion of people completing HCV therapy, adherence in the present analysis was significantly lower among people receiving OST than in those not receiving OST (88% vs 97%). The proportion of people with genotype 1a who received ribavirin was higher among those receiving OST compared with those not receiving OST. As such, it may be possible still that the lower adherence in the OST group was partly driven by the larger proportion of genotype 1a participants who received ribavirin as compared with those not receiving OST.

Although adherence to therapy was lower for people receiving OST, there was no difference in SVR12 between those receiving and not receiving OST. The 94% SVR12 among people who received ombitasvir/paritaprevir/ritonavir and dasabuvir with or without ribavirin is similar to other clinical trials of DAA therapy achieving responses of 92%–96% in people receiving stable OST, with no differences observed between people receiving and not receiving OST [7, 8, 11]. Data from a large registry of real-world data from Germany demonstrated an SVR of 85% (n = 528) in people receiving OST and DAA therapy compared with 91% in those not receiving OST (n = 5582) [27]. The major reason for not attaining SVR was not viral relapse (2% OST vs 4% no OST) but loss to follow-up between the end of treatment and SVR12 (8% OST vs 2% no OST) [27]. This issue of loss to follow-up between end of treatment and SVR12 has also been observed in real-world studies among people with a history of injecting drug use treated with DAA therapies [28–30]. However, given that these individuals completed treatment and attained an end-of-treatment response, it is highly likely that they would have attained an SVR. Differences in loss to follow-up were not observed between the OST and non-OST populations in the present analysis because patients with missing SVR12 data were low in both groups (2% [3/149] and 1% [48/4598], respectively). Treatment was also well tolerated in this study. Collectively, these data provide further support for the integration of DAA therapy in settings that provide co-location of OST services, given the high response to therapy in people stable on OST in this study.

Despite international recommendations that support HCV therapy among people receiving OST [23–26], there are still some clinicians who remain reluctant to provide HCV treatment to people receiving OST based on concerns of adherence, response to therapy, and re-infection. In the United States in 2014, 88% of US state Medicaid committees had restrictions for the reimbursement of therapy for people who have used illicit drugs or alcohol or are receiving OST from DAA-based therapy (irrespective of disease stage) [3]. As such, the data from this study are important to continue to build the evidence base to demonstrate that DAA therapy is effective in people receiving OST.

This study has a number of limitations. First, people with ongoing drug use were excluded from participating in these clinical trials. As such, the enrolled participants represent a selected population likely very engaged in care, and these findings cannot be generalized to other populations of people receiving OST who may be injecting drugs. Participants receiving OST were initially excluded from the phase III program and were evaluated separately in a phase IIb pilot study. People receiving OST were eligible for inclusion in phase III/IIIb studies following preliminary data demonstrating an SVR of 97% in a pilot study of 38 people receiving methadone or buprenorphine and ombitasvir/paritaprevir/ritonavir plus dasabuvir and ribavirin. Second, this is a post hoc analysis of phase III clinical trials, and this study was not specified before starting these clinical trials. Third, given that people receiving OST were less likely to return their pill bottles, a lower proportion of people receiving OST had available data on adherence compared with those not receiving OST (81% vs 91%, respectively). As such, it is possible that adherence to therapy among those on OST was overestimated in this study and there was a larger difference in adherence compared with those not receiving OST. However, irrespective of these limitations, these data are important to publish to add to the body of peer-reviewed evidence demonstrating that people on stable OST can be successfully treated with DAA therapy.

In conclusion, these data demonstrate that ombitasvir/paritaprevir/ritonavir and dasabuvir with or without ribavirin is well tolerated and effective in people receiving OST, despite a lower adherence to therapy compared with people not receiving OST. Further international trials are underway to evaluate DAA therapy in people receiving OST and recent PWID with ombitasvir/paritaprevir/ritonavir and dasabuvir with or without ribavirin (D3FEAT, NCT02498015) and sofosbuvir/velpatasvir for recent PWID (SIMPLIFY, NCT02336139; HERO, NCT02824640); these trials should provide further data in this regard. These data will be crucial for expanding access to testing, care, and treatment for PWID and achieving HCV elimination in this priority population.

## Supplementary Data

Supplementary materials are available at *Open Forum Infectious Diseases* online. Consisting of data provided by the authors to benefit the reader, the posted materials are not copyedited and are the sole responsibility of the authors, so questions or comments should be addressed to the corresponding author.

Supplementary Table
