# Supplementary material for: Efficacy and Safety of Ombitasvir/Paritaprevir/Ritonavir and Dasabuvir With or Without Ribavirin in Patients With Chronic Hepatitis C Virus Genotype 1 Infection Receiving Opioid Substitution Therapy: A Post Hoc Analysis of 12 Clinical Trials
Source: Open Forum Infect Dis. 2018 Sep 27;5(11):ofy248. doi: 10.1093/ofid/ofy248 (PMC6222025; doi:10.1093/ofid/ofy248)
Supplement: Supplementary Table [file ofy248_suppl_supplementary_table.docx]

**Supplementary Table 1.** **Post-baseline laboratory abnormalities among patients with chronic HCV genotype 1 receiving ombitasvir/paritaprevir/ritonavir and dasabuvir with or without ribavirin, stratified by receipt of opioid substitution therapy**

|  | **Receiving OST** | | | **Not receiving OST** | | |
| --- | --- | --- | --- | --- | --- | --- |
|  | **All N=149** | **No RBV N=11** | **+ RBV N=138** | **All N=4598** | **No RBV N=1525** | **+ RBV N=3061** |
| Hemoglobin |  |  |  |  |  |  |
| Grade 3 (<8.0–6.5 g/dL) | 2 (1.3) | 0 | 2 (1.4) | 15 (0.3) | 0 | 15 (0.5) |
| Grade 4 (<6.5 g/dL) | 0 | 0 | 0 | 1 (<0.1) | 0 | 1 (<0.1) |
| ALT |  |  |  |  |  |  |
| Grade 3 (>5–20 × ULN) | 0 | 0 | 0 | 27 (0.6) | 5 (0.3) | 22 (0.7) |
| Grade 4 (>20 × ULN) | 0 | 0 | 0 | 6 (0.1) | 0 | 6 (0.2) |
| AST |  |  |  |  |  |  |
| Grade 3 (>5–20 × ULN) | 0 | 0 | 0 | 16 (0.3) | 4 (0.3) | 12 (0.4) |
| Grade 4 (>20 × ULN) | 0 | 0 | 0 | 1 (<0.1) | 0 | 1 (<0.1) |
| Total bilirubin |  |  |  |  |  |  |
| Grade 3 (>3–10 × ULN) | 8 (5.4) | 0 | 8 (5.8) | 191 (4.2) | 8 (0.5) | 183 (6.0) |
| Grade 4 (>10 × ULN) | 0 | 0 | 0 | 6 (0.1) | 0 | 6 (0.2) |
| ALT, alanine aminotransferase; AST, aspartate aminotransferase; HCV, hepatitis C virus; OST, opioid substitution therapy; RBV, ribavirin; ULN, upper limit of normal.  Grading according to CTCAE v4.03. | | | | | | |
